# Modified facelift incision and superficial musculoaponeurotic system flap in parotid malignancy: a retrospective study and review of the literature

**DOI:** 10.1186/s12957-020-1785-3

**Published:** 2020-01-09

**Authors:** Evangelos I. Giotakis, Aris I. Giotakis

**Affiliations:** First Department of Otorhinolaryngology, Hippocration Hospital, Medical University of Athens, National and Kapodistrian University of Athens, Vas. Sofias 114, 11527 Athens, Greece

**Keywords:** Parotid neoplasms, Rhytidoplasty, Superficial musculoaponeurotic system, Gustatory sweating, Cosmetics

## Abstract

**Background:**

Data reporting the use of modified facelift incision (MFI) approach with or without superficial musculoaponeurotic system (SMAS) reconstruction in parotid malignancy are limited. To enhance the limited knowledge in this subject, the authors of the current study report quality data of MFI in patients with parotid malignancy with or without SMAS reconstruction.

**Methods:**

We performed a retrospective review of parotid malignancy patients treated with the MFI over a 5-year period (2015–2019) in the 1st ENT University Department, University of Athens, Greece.

**Results:**

We identified five patients with parotid malignancy. We performed MFI parotidectomy in 5/5 patients and SMAS reconstruction in 2/5 patients. All tumors were classified as T1N0M0. After a mean follow-up of 43.6 months (minimum, 36; maximum, 55), we noted no recurrence. The patients reported no Frey’s syndrome.

**Conclusions:**

The authors of the current study suggest consideration of the MFI approach in parotid malignancy. A MFI approach should at least favor small parotid tumors without neck metastatic disease (T1cN0). Surgeons could also address larger tumors with a MFI approach. Surgeons should reconstruct the parotid lodge with a SMAS advancement flap in tumors not in proximity with the SMAS.

## Background

The traditional approach to the parotid gland tumors is the bayonet-shaped incision described by Blair. This access is relatively easy to perform and provides a good surgical exposure. However, it is associated with a noticeable scar in the pre-auricular and cervical region, a tissue deficiency in the parotid region with a corresponding postoperative imprint and Frey’s syndrome.

These significant drawbacks led head and neck surgeons to develop various techniques for parotid surgery. In 1967, Appiani introduced the use of facelift incision for tumor excision to avoid the postoperative visible scar [1]. Ten years later, Mitz and coauthors described the use of the superficial musculoaponeurotic system (SMAS) advancement flap in the parotid to avoid the postoperative imprint [2]. Additionally, the SMAS flap also succeeded in reducing the incidence of Frey’s syndrome [3].

In the following years, multiple studies supported the use of the modified facelift incision (MFI) approach and SMAS flap in the treatment of benign parotid tumors. These studies regarded parotid malignancy as a contraindication for the use of MFI and SMAS due to the increased risk of residual disease and recurrence [3–7]. Data supporting otherwise are limited. Few studies have examined MFI and SMAS in parotid malignancy. Terris and coauthors reported excision of two unexpected malignancies with MFI [8]. Charakorn analyzed the use of MFI in parotid tumors including 22% malignant tumors [9]. Other studies suggested that as long as a plane of normal tissue and associated SMAS can be excised along with the tumor and superficial planes are not involved, MFI and/or SMAS are reasonable also for malignancy [10–12]. Lastly, Ambro and coauthors concluded that malignancy should not preclude reconstruction with SMAS flap [13].

To enhance the limited knowledge in this subject and complement the abovementioned studies, the authors of the current study report consistent data of MFI approach in patients with parotid malignancy with or without SMAS reconstruction, such as tumor staging, preoperative diagnostic, parotid approach and reconstruction, acute and late complications, and long-term follow-up.

## Methods

We performed a retrospective review over a 5-year period (2015–2019) in the 1st ENT University Department, University of Athens, Greece. Specifically, we searched for ICD-10 codes D11.0 (benign parotid tumor) and C07 (malignant parotid tumor). After consulting the parotidectomy operative reports, we selected the patients with a MFI approach for parotid malignancy with or without SMAS reconstruction. We recorded the patients’ age, preoperative radiologic examinations, preoperative fine needle aspiration (FNA) findings, surgical approach, reconstruction, histology, tumor margins, postoperative complications such as temporary or permanent facial nerve palsy, hematoma and salivary fistula, tumor staging, adjuvant treatment, follow-up, and occurrence of Frey’s syndrome. The study was approved by the Institutional Ethics Committee of the National and Kapodistrian University of Athens.

## Results

During the study period, 22 patients received a MFI parotidectomy. Among them, we identified five patients with parotid malignancy.

The following information applies for all patients unless mentioned otherwise. All patients received preoperatively an otorhinolaryngology–head and neck examination and magnetic resonance imaging (MRI) of the head and neck region. All tumors were detected in the lower lobe, three in the left lower lobe. There was neither clinical nor radiologic suspicion of malignancy. We performed ultrasonography (US)-guided FNA in 4/5 patients. Postoperatively, a pathologist diagnosed malignancy. We completed tumor staging with a contrast thorax/abdomen computer tomography (CT). All tumors were resected with clear margins (> 8 mm) and were classified as T1N0M0R0. After consultation with the multidisciplinary tumor board (MDT), we performed no adjuvant treatments. In routine follow-up visits, we included regularly neck US and yearly contrast thorax/abdomen CT scan. Until September 2019 (minimum 3 years after diagnosis), we observed no tumor recurrence.

To avoid unnecessary repetition and to highlight the differences between MFI with SMAS and MFI without SMAS, we present the operative steps in cases 2 and 3. The main data of all five cases are presented in Tables 1 and 2.

**Table 1:** Epidemiologic characteristics and preoperative examinations of all cases

Case	1	2	3	4	5
Surgery	02.2015	10.2015	03.2016	08.2016	09.2016
Gender	Female	Female	Female	Female	Male
Age	27	53	62	65	48
MRI^†^	Pleomorphic^‡^	Cystadenolymphoma	Pleomorphic^‡^	Pleomorphic^‡^	Cystadenolymphoma
FNA^§^	Refused	Benign tumor cells	Malignancy suspicion	Benign tumor cells	Malignancy suspicion

^†^Magnetic resonance imaging

^‡^Adenoma

^§^Fine needle aspiration

**Table 2:** Parotid reconstruction, histology, staging, and postoperative follow-up of all cases

Case	1	2	3	4	5
SMAS†	Yes	No	Yes	No	No
Carcinoma	Acinic cell^‡^	Mucoepidermoid^‡^	Mucoepidermoid^‡^	Acinic cell^‡^	Myoepithelial^‡^
cTNM	T1N0M0	T1N0M0	T1N0M0	T1N0M0	T1N0M0
Complications	None	None	None	Temporary facial paralysis*	None
Follow-up^§^	55	48	42	37	36
Recurrence	No	No	No	No	No
Frey’s syndrome	No	No	No	No	No

^†^Superficial musculoaponeurotic system

^‡^Low-grade

^§^In months

*House-Brackmann III

### Case 2

**Fig. 1:**
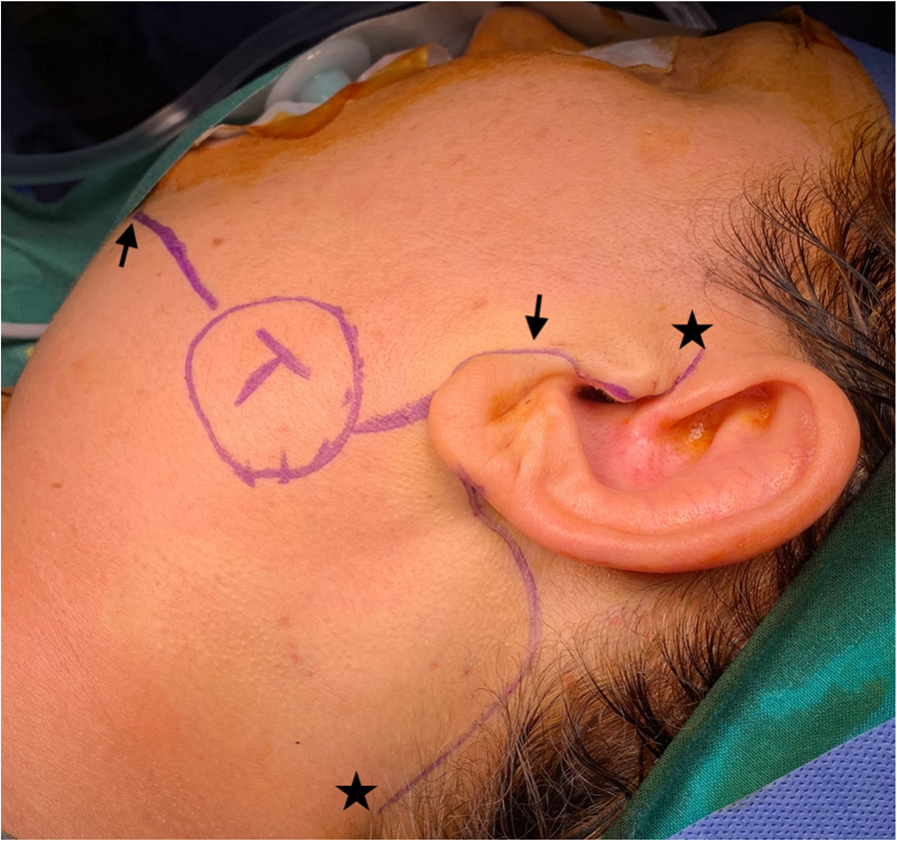
Case 2. The tumor is marked with a T and a circle. The anterior line outlines the mandible from the mentum (anterior arrow) to the temporomandibular joint (posterior arrow). The posterior line outlines the modified facelift incision from the tragus to the hair line (from the cranial to the caudal star)

**Fig. 2:**
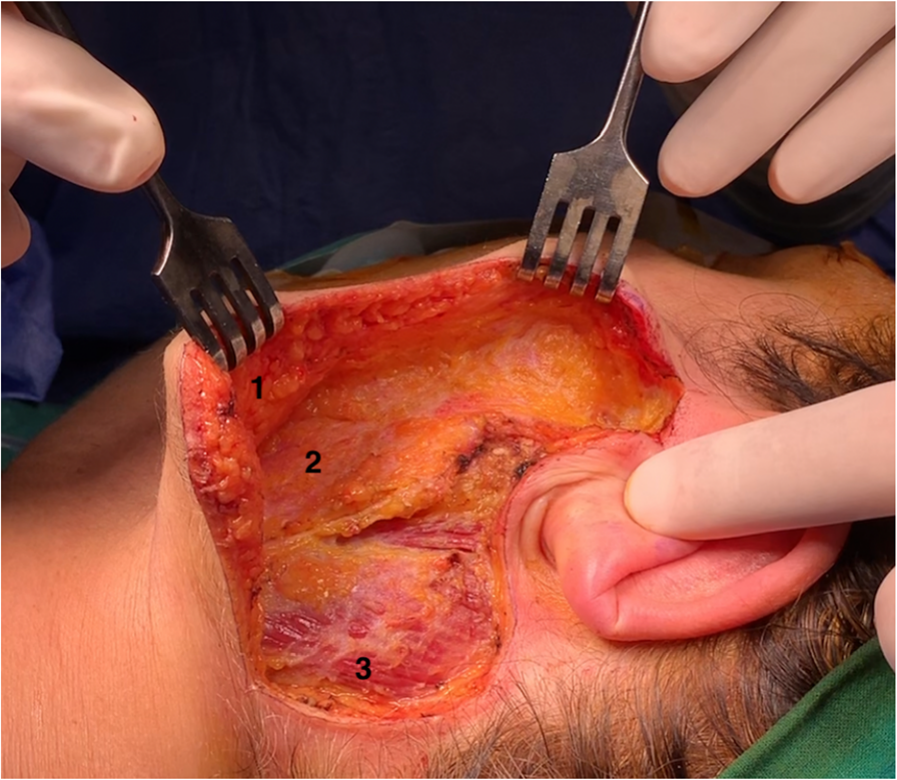
Case 2. After left superficial parotidectomy with modified facelift incision. Number 1 indicates the thick skin flap, number 2 the superficial musculoaponeurotic system, and number 3 the sternocleidomastoideus muscle

In October 2015, a 53-year-old female patient presented with a left superficial parotid tumor of the lower lobe. Radiologic examinations revealed a cystadenolymphoma. FNA showed benign tumor cells, suspicious for cystadenolymphoma. We performed a MFI superficial parotidectomy with a thick skin flap without SMAS reconstruction (Figs. 1 and 2). Specifically, after the MFI, the skin flap is elevated on the plane of the parotid fascia. Superficial parotidectomy follows as usual. Postoperatively, we observed no complications. A pathologist diagnosed a low-grade mucoepidermoid carcinoma. The patient noted no gustatory sweating after 48 months of follow-up.

### Case 3

**Fig. 3:**
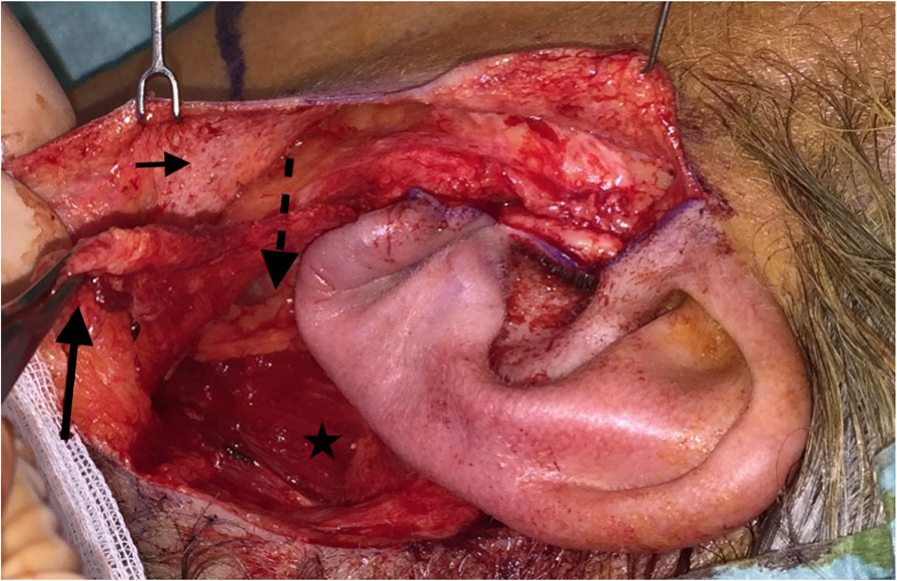
Case 3. After left superficial parotidectomy with modified facelift incision. Dissection of the skin flap (small arrow) and superficial musculoaponeurotic system flap (long thick arrow). The intermittent arrow and the star indicate the parotid capsule and the sternocleidomastoideus muscle respectively

**Fig. 4:**
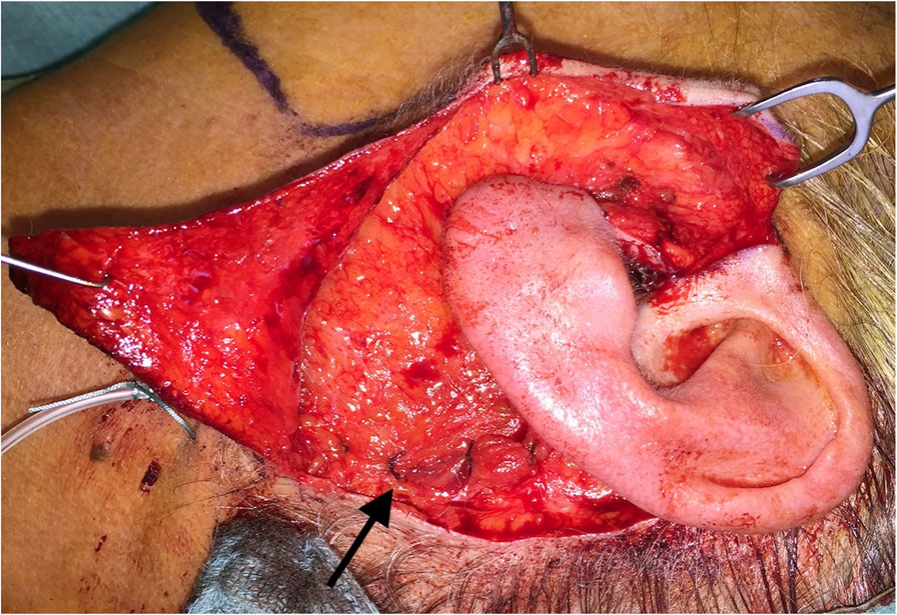
Case 3. After reconstruction of the postoperative imprint with the superficial musculoaponeurotic system advancement flap. The arrow indicates one of the sutures

In March 2016, a 62-year-old female patient presented with a left superficial parotid tumor of the lower lobe. Radiologic examinations revealed a pleomorphic adenoma. FNA showed suspicion for malignancy. We performed a MFI superficial parotidectomy with SMAS reconstruction (Figs. 3 and 4). Specifically, after the MFI, the first superficial skin flap is elevated. The first superficial skin flap includes the skin and 1–2 mm subcutaneous tissue. The whole flap region is injected with 1 ml of 1:200000 Adrenalin with 2% Xylocain diluted in 9 ml NaCl to minimize bleeding. Then, the second deeper flap (SMAS flap) is raised including all tissue till the parotid fascia. The SMAS flap is cranially incised horizontally 1 cm below the zygomatic arch and then vertically along the posterior border of the platysma muscle. After superficial parotidectomy, the SMAS flap is repositioned with 3/0 Vicryl sutures on the medial border of sternocleidomastoid muscle. Care is taken not to apply much or insufficient tension while repositioning the SMAS flap to avoid facial asymmetry or the postoperative imprint respectively. Postoperatively, we observed no complications. A pathologist diagnosed a low-grade mucoepidermoid carcinoma. The patient noted no gustatory sweating after 42 months of follow-up.

## Discussion

Data reporting the use of MFI approach with or without SMAS reconstruction in parotid malignancy are limited. Mostly, such data are included as incidental findings in MFI studies for benign parotid tumors. Moreover, most studies specifically exclude patients with suspected malignancy in clinical, radiologic, and/or FNA examination [14–18]. In a systematic review, Grover and D’Souza searched for MFI in parotidectomy and identified 11 relevant studies matching their selection criteria [7]. The authors assessed 628 patients and detected 46 (7.4%) parotid malignancies. Among them, 25 mucoepidermoid carcinomas, 11 adenoid cystic carcinomas, and 10 squamous cell carcinomas. While 46 parotid malignancies could provide much information, these studies failed to mention data such as tumor size, other tumor characteristics, long-term follow-up, and complications [8, 9]. To enhance the limited knowledge in this subject, the authors of the current study report quality data of MFI in patients with parotid malignancy with or without SMAS reconstruction.

We present five patients with parotid malignancy treated with MFI. Preoperatively, all patients received head and neck MRI. The radiologists detected no signs of malignancy and no cervical lymph nodes. All tumors were located superficial in the lower lobe and were smaller than 2 cm. We performed FNA in 4/5 patients. Case 1 refused FNA examination. FNA showed suspicion for malignancy in 2/4 patients.

In malignancy, it seems reasonable to perform a Blair’s incision to extend the incision to the neck if a neck dissection is indicated. This could be helpful in order to address the neck by cN+ or by larger parotid tumors to resect possible occult neck metastases. Qian and coauthors performed elective neck dissection in 84 patients with cN0 salivary gland carcinoma. They detected occult lymph node metastasis in 8/84 (9.5%) patients. The authors concluded that elective neck dissection has a limited role in cN0 salivary gland carcinoma [19]. Our case series included five T1cN0 patients. Occult neck metastases present more frequently in advanced tumor stage and tumor size [20], in facial nerve paralysis, extraparotideal extension or high tumor grade [21, 22], and in more than 50% of the cases in anaplastic carcinoma, squamous cell carcinoma, and salivary duct carcinoma [21, 23, 24]. However, occult metastases are also detected in low-grade carcinomas and T1 and T2 carcinomas [23, 25]. These data could justify our decision to avoid a neck dissection by T1cN0 parotid tumors without clinical signs of malignancy.

On the contrary, Zbären and coauthors suggested the routine elective neck dissection in patients with cN0 parotid malignancy [25]. In their retrospective study, the authors assessed the outcome of 83 cN0 patients with preoperatively known malignancy divided in two groups; one group with an elective neck dissection and one observation group without elective neck dissection. The authors reported that all the seven neck recurrences occurred in the observation group. Similarly, Kawata and coauthors recommended the elective neck dissection in primary parotid cN0 carcinoma due to the low accuracy of preoperative diagnosis [26]. However, they also reported that elective neck dissection may not be necessary in low-grade carcinoma. In their systematic review, Valstar and coauthors concluded that neck treatment should be performed, either with elective neck dissection or with adjuvant radiotherapy [27].

It seems that whether to perform an elective neck dissection in cN0 parotid malignancy is a matter of debate. While an elective neck dissection is certainly the safer way, the data could also justify the avoidance of an elective neck dissection under the certain criteria described above, especially in T1 tumors. In our case series of T1cN0, we observed no recurrence after a mean follow-up of 43.6 months (minimum, 36; maximum, 55). Nevertheless, our decision to avoid a neck dissection was based clearly on oncologic criteria. It should not be associated with our decision to perform the superficial parotidectomies with a MFI approach.

In our case series, we suspected malignancy in 2/5 patients. It is common knowledge that parotid malignancy should be preferably treated with a total parotidectomy, if the facial nerve shows no signs of tumor invasion. In our case series, tumor size was smaller than 2 cm in all patients and no patient showed either clinical or radiologic signs of malignancy. In the two patients with suspected malignancy, a superficial parotidectomy was considered sufficient for tumor control due to the small tumor size and tumor location. After histologic confirmation of malignancy, we did not perform a total parotidectomy. We based this decision on the clear tumor margins, the tumor histologic types, and the increased incidence of permanent facial nerve palsy by revision [28, 29]. Moreover, our decision to perform a superficial parotidectomy should not be associated with our decision to perform the MFI. A total parotidectomy is also possible through MFI approach as Nouraei and coauthors described. The authors compared the MFI and the Blair’s incision in cadavers. They concluded that the control and the visibility of the operating field is the same when using the MFI and the Blair’s incision [30].

The abovementioned data could point out the neck dissection as the main contraindication of MFI approach in parotid malignancy. If the disease requires a neck dissection incision to address the neck, it seems reasonable to elongate the already-performed Blair’s incision. However, surgeons could in theory convert the MFI in a classic neck dissection incision or perform the neck dissection incision separate from the MFI. Obviously, such data are poor. But if the surgeon is confident enough to perform this approach, without jeopardizing oncologic safety, then this should not be considered prohibitive. Such an approach could improve the esthetic result, since multiple studies described the esthetic superiority of MFI compared to the Blair’s incision [16, 31, 32]. Interestingly, Shin and coauthors described feasible robotic selective neck dissection through a MFI in parotid cancer [33].

In our case series with five T1cN0 patients, we achieved adequate tumor control with a MFI superficial parotidectomy without neck dissection. In 2/5 patients, we performed reconstruction with a SMAS-advancement flap. Several studies described the main advantages of the SMAS flap. These include the avoidance of the postoperative imprint and the reduction in the incidence of Frey’s syndrome [2, 4, 6, 31, 34–36].

Paris and coauthors assessed the postoperative incidence of Frey’s syndrome in ten patients undergoing parotidectomy with SMAS flap. After a mean follow-up of 16 months, no patient reported signs of Frey’s syndrome [31]. In their prospective study, Wille-Bischofberger and coauthors studied the incidence of Frey’s syndrome and the cosmetic result after parotidectomy. The authors compared two groups: 23 patients without SMAS flap and 25 patients with SMAS flap. After 23 months, the incidence of Frey’s syndrome was 43% for the non-SMAS group and 0% for the SMAS group (*p* = 0.003). Interestingly, after 78 months at final follow-up, the incidence of Frey’s syndrome was 41% for the non-SMAS group and 56% for the SMAS group (*p* > 0.2). Nevertheless, the rate of the satisfactory cosmetic result was 35% in the non-SMAS group and 96% in the SMAS group (*p* < 0.05) [34]. Also, in their meta-analysis, Dulguerov and coauthors reported that the SMAS flap was associated with a decrease of clinical Frey’s syndrome with an odds ratio of 0.42 (confidence interval 0.32–0.56) [35].

Despite its advantages, we performed the SMAS flap only in 2/5 patients. By suspicion of malignancy or in parotid tumors in proximity with the SMAS, surgeons must carefully indicate and perform a SMAS flap. The dissection of the SMAS flap might jeopardize oncologic safety, as it is usually adjacent to the parotid capsule. In their study, Meningaud and coauthors concluded that the SMAS flap might possibly appear to offer a new standard procedure for parotidectomy, except for malignant tumors [16]. In our case series, the decision to avoid a SMAS flap correlated mainly to the close tumor proximity with the SMAS in case 2, 4, and 5. If SMAS flap is contraindicated, surgeons should perform a thick skin flap [36]. After a mean follow-up of 43.6 months (minimum, 36; maximum, 55), no patient of our case series reported sings of Frey’s syndrome. Moreover, we noted no complication except temporary facial nerve palsy by 1/5 patients. Grover and coauthors studied the safety of the facelift incision in 628 patients. The authors concluded that the complication rates with MFI approach were not increased compared to the Blair’s incision [7].

## Conclusions

The authors of the current study suggest consideration of the MFI approach in parotid malignancy. A MFI approach should at least favor small parotid tumors without neck metastatic disease (T1cN0). Surgeons could also address larger tumors with a MFI approach. Most importantly, high-grade tumors and tumors with higher probability of occult neck metastatic disease require great caution. Surgeons should reconstruct the parotid lodge with a SMAS advancement flap in tumors not in proximity with the SMAS. In no means should surgeons endanger the oncologic result of surgery for esthetic and/or functional reasons.

## Data Availability

All data generated or analysed during this study are included in this published article.

## Abbreviations

CT: Computer tomography
FNA: Fine needle aspiration
MDT: Multidisciplinary tumor board
MFI: Modified facelift incision
MRI: Magnetic resonance imaging
SMAS: Superficial musculoaponeurotic system
US: Ultrasonography

## References

[1] Appiani E (1967). Handling of a parotidectomy and muscular graft. Prensa Med Argent..

[2] Mitz V, Peyronie M (1976). The superficial musculo-aponeurotic system (SMAS) in the parotid and cheek area. Plast Reconstr Surg..

[3] Allison GR, Rappaport I (1993). Prevention of Frey’s syndrome with superficial musculoaponeurotic system interposition. Am J Surg..

[4] Barbera R, Castillo F, D'Oleo C, Benitez S, Cobeta I (2014). Superficial musculoaponeurotic system flap in partial parotidectomy and clinical and subclinical Frey’s syndrome. Cosmesis and quality of life. Head Neck..

[5] de Vicente JC, Gonzalez-Garcia M, de Villalain L, Fernandez-Valle A (2015). Modified facelift approach combined with a superficial musculoaponeurotic system flap in the treatment of benign parotid tumors. J Craniomaxillofac Surg..

[6] Dell'Aversana Orabona G, Salzano G, Abbate V, Piombino P, Astarita F, Iaconetta G (2015). Use of the SMAS flap for reconstruction of the parotid lodge. Acta Otorhinolaryngol Ital..

[7] Grover N, D'Souza A (2013). Facelift approach for parotidectomy: an evolving aesthetic technique. Otolaryngol Head Neck Surg..

[8] Terris DJ, Tuffo KM, Fee WE (1994). Modified facelift incision for parotidectomy. J Laryngol Otol..

[9] Charakorn C (1998). The scarless rhytidectomy incision in superficial parotidectomy. Journal of the Medical Association of Thailand =. Chotmaihet thangphaet..

[10] Murthy P, Hussain A, McLay KA (1997). Parotidectomy through a rhytidectomy incision. Clin Otolaryngol Allied Sci..

[11] Honig JF (2004). Facelift approach with a hybrid SMAS rotation advancement flap in parotidectomy for prevention of scars and contour deficiency affecting the neck and sweat secretion of the cheek. J Craniofac Surg..

[12] Jost G, Guenon P, Gentil S (1999). Parotidectomy: a plastic approach. Aesthetic Plast Surg..

[13] Ambro BT, Goodstein LA, Morales RE, Taylor RJ (2013). Evaluation of superficial musculoaponeurotic system flap and fat graft outcomes for benign and malignant parotid disease. Otolaryngol Head Neck Surg..

[14] Foustanos A, Zavrides H (2007). Face-lift approach combined with a superficial musculoaponeurotic system advancement flap in parotidectomy. Br J Oral Maxillofac Surg..

[15] Pang PC, Chu GM, To EW (2000). The use of modified rhytidectomy for parotidectomy. Br J Plast Surg..

[16] Meningaud JP, Bertolus C, Bertrand JC (2006). Parotidectomy: assessment of a surgical technique including facelift incision and SMAS advancement. J Craniomaxillofac Surg..

[17] Lohuis PJ, Tan ML, Bonte K, van den Brekel MW, Balm AJ, Vermeersch HB (2009). Superficial parotidectomy via facelift incision. Ann Otol Rhinol Laryngol..

[18] Lee SY, Koh YW, Kim BG, Hong HJ, Jeong JH, Choi EC (2011). The extended indication of parotidectomy using the modified facelift incision in benign lesions: retrospective analysis of a single institution. World J Surg..

[19] Qian K, Guo K, Zheng X, Sun W, Sun T, Chen L (2019). The limited role of elective neck dissection in patients with cN0 salivary gland carcinoma. J Craniomaxillofac Surg..

[20] Armstrong JG, Harrison LB, Thaler HT, Friedlander-Klar H, Fass DE, Zelefsky MJ (1992). The indications for elective treatment of the neck in cancer of the major salivary glands. Cancer..

[21] Frankenthaler RA, Byers RM, Luna MA, Callender DL, Wolf P, Goepfert H (1993). Predicting occult lymph node metastasis in parotid cancer. Archives of otolaryngology--head & neck surgery..

[22] Ferlito A, Pellitteri PK, Robbins KT, Shaha AR, Kowalski LP, Silver CE (2002). Management of the neck in cancer of the major salivary glands, thyroid and parathyroid glands. Acta Otolaryngol..

[23] Stennert E, Kisner D, Jungehuelsing M, Guntinas-Lichius O, Schroder U, Eckel HE (2003). High incidence of lymph node metastasis in major salivary gland cancer. Archives of otolaryngology--head & neck surgery..

[24] Harish K (2004). Management of primary malignant epithelial parotid tumors. Surg Oncol..

[25] Zbaren P, Schupbach J, Nuyens M, Stauffer E (2005). Elective neck dissection versus observation in primary parotid carcinoma. Otolaryngol Head Neck Surg..

[26] Kawata R, Koutetsu L, Yoshimura K, Nishikawa S, Takenaka H (2010). Indication for elective neck dissection for N0 carcinoma of the parotid gland: a single institution’s 20-year experience. Acta Otolaryngol..

[27] Valstar MH, van den Brekel MW, Smeele LE (2010). Interpretation of treatment outcome in the clinically node-negative neck in primary parotid carcinoma: a systematic review of the literature. Head Neck..

[28] Bittar RF, Ferraro HP, Ribas MH, Lehn CN (2016). Facial paralysis after superficial parotidectomy: analysis of possible predictors of this complication. Braz J Otorhinolaryngol..

[29] Mehle ME, Kraus DH, Wood BG, Benninger MS, Eliachar I, Levine HL (1993). Facial nerve morbidity following parotid surgery for benign disease: the Cleveland Clinic Foundation experience. The Laryngoscope..

[30] Nouraei SA, Al-Yaghchi C, Ahmed J, Kirkpatrick N, Mansuri S, Singh A (2006). An anatomical comparison of Blair and facelift incisions for parotid surgery. Clin Otolaryngol..

[31] Paris J, Richard O, Lafont B, Facon F, Bruzzo M. [Aesthetic parotidectomy: face lift incision and SMAS flap]. Rev Laryngol Otol Rhinol (Bord). 2007;128(4):261-4. 18320934

[32] Bulut OC, Plinkert P, Federspil PA (2016). Modified facelift incision for partial parotidectomy versus bayonet-shaped incision: a comparison using visual analog scale. Eur Arch Otorhinolaryngol..

[33] Shin YS, Choi EC, Kim CH, Koh YW (2014). Robot-assisted selective neck dissection combined with facelift parotidectomy in parotid cancer. Head Neck..

[34] Wille-Bischofberger A, Rajan GP, Linder TE, Schmid S (2007). Impact of the SMAS on Frey’s syndrome after parotid surgery: a prospective, long-term study. Plast Reconstr Surg..

[35] Dulguerov N, Makni A, Dulguerov P (2016). The superficial musculoaponeurotic system flap in the prevention of Frey syndrome: a meta-analysis. The Laryngoscope..

[36] Durgut O, Basut O, Demir UL, Ozmen OA, Kasapoglu F, Coskun H (2013). Association between skin flap thickness and Frey’s syndrome in parotid surgery. Head Neck..

